# Usefulness of Galectin-3 as a Biochemical Marker to Detect Ventricular and Supraventricular Arrhythmias in Children

**DOI:** 10.3390/cimb46100669

**Published:** 2024-10-10

**Authors:** Ewa Moric-Janiszewska, Joanna Wawszczyk, Aleksandra Morka, Małgorzata Kapral

**Affiliations:** 1 Department of Biochemistry, Faculty of Pharmaceutical Sciences in Sosnowiec, Medical University of Silesia in Katowice, Jedności 8B, 41-200 Sosnowiec, Poland; jwawszczyk@sum.edu.pl (J.W.); mkapral@sum.edu.pl (M.K.); 2 Department of Pediatric Cardiology, Faculty of Medical Sciences in Katowice, Medical University of Silesia in Katowice, Medyków 16, 40-752 Katowice, Poland; aleksandra.morka@sum.edu.pl

**Keywords:** ventricular/supraventricular arrhythmia, galectin-3, ELISA analysis, pediatric patients

## Abstract

Galectin-3 (Gal-3) has been demonstrated to play a pivotal role in the pathogenesis of several fibrotic disorders. A number of studies have examined the relationship between galectin-3 levels and cardiac fibrosis in heart failure. Nevertheless, the role of galectin-3 in the etiology of supraventricular (SVa) and ventricular (Va) arrhythmias remains largely unexamined. The objective of this prospective study was to investigate the potential correlation between galectin concentration and the occurrence of idiopathic cardiac arrhythmias in pediatric patients. Biochemistry analysis was performed on 30 children (11–18 years; 14 boys and 16 girls). The control group consisted of 20 children. Cardiac arrhythmia was confirmed by a 24 h Holter ECG recording. Serum galectin-3 levels were measured via enzyme-linked immunosorbent assay (ELISA). Statistical analysis of the data showed significant associations between creatinine kinase (CK) and Gal-3 in patients with SVa (SVT—supraventricular tachycardia) arrhythmias, suggesting a potential effect of CK on Gal-3 levels. However, no correlation was identified between Gal-3 concentration and the occurrence of cardiac arrhythmias under investigation. We concluded that galectin-3 does not have the potential to be a biomarker in the diagnosis of idiopathic arrhythmias in pediatric patients.

## 1. Introduction

**Figure 1:**
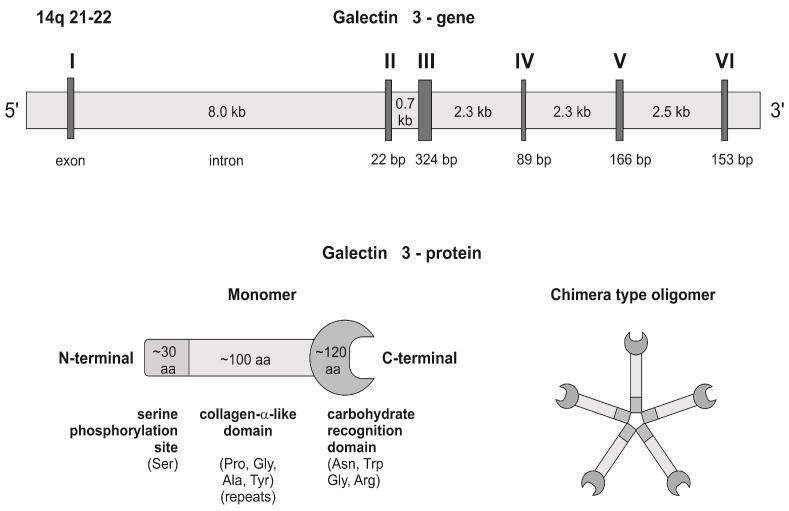
The genomic organization of the human galectin-3 gene and the structure of the galectin-3 protein.

Atrial fibrillation (AF) is an extremely low prevalence in pediatric patients. However, supplementary supraventricular (SVEs) and ventricular (VEs) extrasystoles, as well as supraventricular (SVT) and ventricular (VT) tachycardia are far more common. They represent a significant health concern, particularly in infants, due to their capacity to precipitate cardiovascular collapse and a spectrum of grave complications, some of which may prove fatal [1,2]. The underlying mechanisms of arrhythmias are broadly classified into the following categories: atrioventricular reentry tachycardia, atrioventricular nodal reentry tachycardia, twin atrioventricular nodal tachycardia, macroreentrant atrial tachycardia, focal atrial tachycardia, ventricular ectopy, and ventricular tachycardia [2,3]. Galectin-3 is a protein classified within the β-galactoside-binding lectin family, which has the ability to form a complex with matrix proteins, including cell surface receptors, collagen, elastin, and fibronectin [4]. This protein is present in a multitude of cell and tissue types, where it plays a crucial role in a number of biological processes, including cell adhesion, activation, growth, differentiation, and apoptosis [5]. Galectin-3 has been demonstrated to serve as a mediator of both cardiac inflammation and fibrosis, promoting the transformation of fibroblasts into “active” myofibroblasts and consequently enhancing the production and secretion of matrix proteins implicated in fibrogenesis [6]. Recently, galectin-3 has also been identified as a biomarker of risk stratification and prognosis in heart failure patients. The functional role of circulating galectin-3 in the context of atrial fibrillation remains to be fully elucidated [7]. Previous research has demonstrated that elevated galectin-3 levels are predictive of incident atrial fibrillation [8] and correlate with the extent of atrial fibrosis [9]. To date, 15 different mammalian galectins have been characterized, all of which contain a conserved carbohydrate-recognition-binding domain (CRD) comprising approximately 130 amino acids. Molecule Gal-3 shows a domain structure. The galectin family is subdivided into three subgroups, distinguished by the amount and structure of their CRDs: the prototype group, the chimera group, and the tandem repeat group [8]. Galectin-3, is the sole vertebrate chimera-type galectin, and contains one CRD linked to an unconventionally lengthy N-terminal proline- and glycine-rich region [10,11,12] (Figure 1). Human galectin-3 is a 35-kDa protein that is encoded by *LGALS3* gene, which is mapped to chromosome 14 in a region between positions q21 and q22. This gene is constituted by six exons and five introns, which, together, span approximately 17 kilobases. Exons IV–VI are responsible for encoding the C-terminal domain, which contains the CRD domain. Exon III and 18 base pairs of exon II are responsible for encoding a long and flexible N-terminal domain, which includes sites for serine phosphorylation and other crucial determinants for the alternative secretion pathway of the protein [11,12] (Figure 1).

Gal-3 has no signaling sequence, and as such, is mainly located in the cytoplasm. Gal-3 expression has been demonstrated in many cell types, including neutrophils, macrophages, mast cells, fibroblasts, osteoclasts, and cancer cells. In biological tissues and organs, the highest concentration of Gal-3 is found in the lungs, spleen, stomach, large intestine, adrenal glands, uterus, and ovaries [10,11,12,13]. This study aimed to determine whether 30 children with ventricular and supraventricular arrhythmias would exhibit elevated galectin-3 concentrations in blood serum compared to a control group of 20 healthy children, as well as to answer the question of whether galectin-3 can be a predictive marker in the diagnosis of idiopathic arrhythmia in children. In this study, the “zero” hypothesis assumed that there is a positive correlation between galectin concentration and cardiac arrhythmias in children.

## 2. Materials and Methods

### 2.1. Research Population

The whole study population comprised 50 children. The main group consisted of 30 patients suffering from supraventricular (11) or ventricular tachycardia (19); 14 were boys and 16 were girls. Cardiac arrhythmia was confirmed via a 24 h Holter ECG recording. The remaining 20 healthy children formed the control group (12 girls and 8 boys). The presence of arrhythmia was excluded through the use of Holter ECG monitoring. The majority of the control group were children who had been conveyed to the emergency department due to hypertension, chest pain, and fainting. The causes of these ailments were unrelated to the heart. The patients ranged in age from 11 to 18 years, with an average age of 15.5 years. All children underwent a series of basic tests, including blood tests, an electrocardiogram (ECG), and blood pressure measurements. All children from study and control groups were diagnosed at the Cardiology Department of the Pediatric Hospital in Katowice. This is a continuation of our research on “Epigenetic, pharmacogenetic, molecular aspects of arrhythmogenic heart disease in children and adolescents” [2,14].

### 2.2. Ethical Issues

“The investigational protocol was approved by the ethics committee of the Medical University of Silesia (PCN/CBN/0052/KB1/142/I/17/18/22). Prior to their participation, the parents or guardians of the underage participants provided signed informed consent, and it was ensured that all participants fully understood and supported the study [2,14]”.

### 2.3. Serum Collection and Storage

Blood samples for galectin-3 ELISA analysis were obtained and immediately processed at the Department of Pediatric Cardiology, Upper Silesian Child Health Centre, in Katowice. The samples underwent centrifugation and were stored at −80 °C until assayed.

### 2.4. Measurement of Galectin-3 Concentration

Serum galectin-3 concentrations were quantified using a RayBio Human Galectin-3 ELISA Kit (RayBiotech Peachtree Corners, GA, USA), a kit that employs an enzyme-linked immunosorbent assay (ELISA) technique. This particular assay employs a specific antibody for human galectin-3, which is coated on a 96-well plate. The absorbance was then measured using a Labtech LT-5000 multiplate reader (Labtech International, Heathfield, UK) at wavelength λ = 450 nm. The level of galectin-3 was evaluated in comparison with the standard curve (a serial dilution of a 200 ng/mL Human Galectin-3 Standard Protein solution), which was obtained under identical experimental conditions. The concentration of galectin-3 in serum samples were determined via interpolation and expressed in ng/mL.

### 2.5. Statistical Analysis

The statistical analysis was carried out using the Statistica PL 13.0 software (StatSoft, Kraków, Poland). The data were subjected to statistical analysis in order to determine any statistically significant differences between the study group and the control group, as well as between the sexes, age groups, and arrhythmia types (i.e., ventricular and supraventricular arrhythmias) in each of the aforementioned subgroups. Given that the clinical variables were distributed normally, the parametric Student’s *t*-test was applied in the statistical analysis. The descriptive statistics for the clinical parameters were demonstrated as arithmetic means and their standard deviations (SD). Given that galectin levels lacked a normal distribution, the non-parametric Mann–Whitney U-test was employed for comparative analyses between groups, with the results presented as medians (Me) and quartiles (Q 25–75%). Statistical analyses were conducted for two independents groups, with a statistical significance threshold of *p* < 0.05. The relationship between the female-to-male ratio was determined through the application of the Chi-square test. Pearson’s correlation was employed to investigate the relationship between the selected variables.

## 3. Results

### 3.1. Patient Characteristics

The study group involved 30 children with cardiac arrhythmia. Ventricular arrhythmia (Va) was observed in 19 patients (63.3%), including 13 with ventricular extrasystoles (VEs) and 6 with ventricular tachycardia (VT). Supraventricular arrhythmias (SVa) manifested in 11 patients, including 8 with supraventricular tachycardia (SVT) and 3 with supraventricular extrasystoles (SVEs). Most patients (23 out of 30) had symptoms of palpitations; 4 described chest pain, and weakness was a common episode (five cases). The patients also felt dizzy and short of breath. The described study group included children who suffered from several symptoms and ailments at the same time. As shown in Table 1, analysis of the 24 h Holter ECG in the study group showed that cardiac arrhythmias occurred in all of them and abnormal beats in the recordings averaged 5.1% (min. 1%, max. 30%). The control group consisted of 20 healthy children, of whom 12 were girls and 8 were boys. The control group was well matched for basic parameters, such as age, sex, height, weight, BSA, and BMI, and was not statistically significantly different from the study group, allowing for comparative analysis. Fainting was observed in 14 individuals from this participant group, of whom 2 were diagnosed with syncope, classified as cardiodepressive syncope. Among adolescents in this group, seven participants experienced chest pain and three exhibited hypertension. Additionally, one instance of nosebleed was observed. Some individuals in the control group had several of the mentioned symptoms and complaints simultaneously. These ailments were found to have no cardiac etiology. The plasma ion (Na^+^, K^+^, Cl^−^, Mg^2+^, Ca^2+^) concentration values for all children were within the normal range. No notable discrepancies were observed between the study group and the control group with regard to the evaluated fundamental clinical, biochemical, and echocardiographic parameters (please see Table 1 for details (modified from [2,14])).

### 3.2. Galectin-3 Levels in Va and SVa Patients

**Figure 2:**
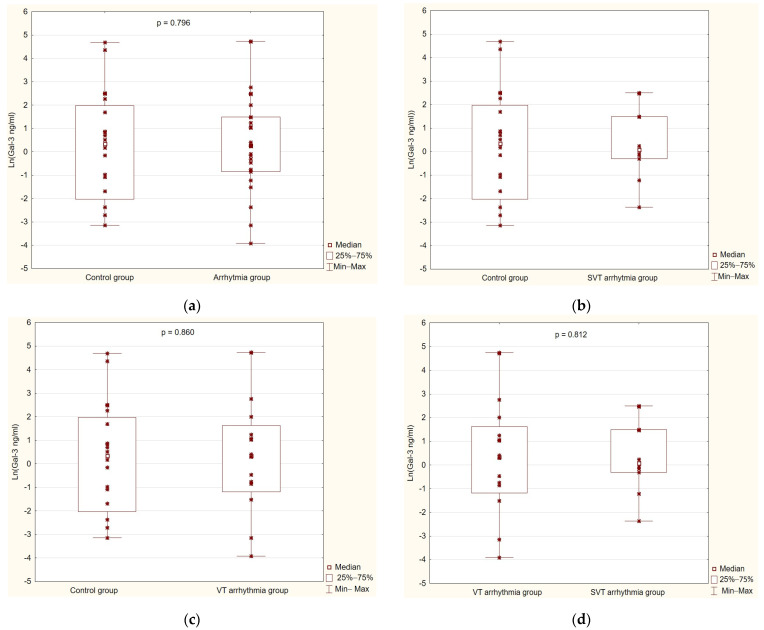
(**a**) A comparison between galectin-3 levels in the control (*n* = 20) and arrhythmia groups (*n* = 26); (**b**) a comparison between galectin-3 levels in the control (*n* = 20) and SVT arrhythmia groups (*n* = 10); (**c**) a comparison between galectin-3 levels in the control (*n* = 20) and VT arrhythmia groups (*n* = 16); (**d**) a comparison between galectin-3 levels in the VT (*n* = 16) and SVT arrhythmia groups (*n* = 10).

During the research, hypotheses were formulated regarding the influence of galectin-3 on the ability to determine the type of cardiac arrhythmia and its progression, as well as other factors that were examined. They were assessed to eliminate or confirm the relationship between the level of galectin-3 and such factors as age, gender, basic cardiac and laboratory parameters, and echocardiographic assessment of the morphology and function of the left ventricle. The obtained concentrations of galectin-3 [ng/mL] were compared between the groups depending on the type of arrhythmia (Figure 2).

The average value in the control group was 1.43 ng/mL, while in arrhythmia group, it was 1.30 ng/mL (Me). Based on the obtained results, it was confirmed that the occurrence of arrhythmia in the study group (all patients) is not related to the concentration of galectin-3 (*p* = 0.798) (Figure 2a). Afterwards, galectin-3 concentrations were compared depending on the type of arrhythmia (SVT 1.10; VT 1.42 ng/mL (Me)). There was no relationship between the type of arrhythmia (SVT/VT) and the concentration of this protein in the study group of patients (*p* = 0.808; *p* = 0.860) (Figure 2b,c). Further, no significant differences were observed in the galectin-3 concentration between the group of patients with VT and patients with SVT (*p* = 0.812) (Figure 2d).

Additionally, the influence of gender on the level of galectin-3 was analyzed regardless of the type of arrhythmia (all patients). The value of coefficient *p* = 0.389 indicates that the level of galectin-3 is not dependent on the gender of the examined patients/children. When the study subjects were divided according to the type of arrhythmia, there was also no statistically significant effect of gender on the level of galectin-3 (SVT—*p* = 0.476; VT—*p* = 0.680).

The statistical analyses performed did not demonstrate the influence of age, oxygen saturation, and electrocardiographic parameters—heart rate (HR), total QRS, and echocardiographic parameters (LVIDD, LVIDS, EF LV, and SF LV)—on the level of this protein.

**Figure 3:**
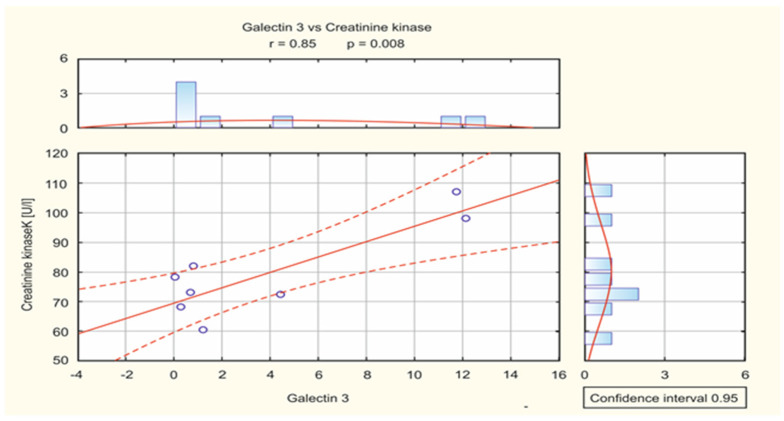
A scatterplot showing galectin-3 concentration in patients with SVT arrhythmia in relation to CK.

When it comes to analyzing the relationship between biochemical parameters, such as the concentration of creatine kinase (CK) and its CK-MB isoenzyme, as well as the CRP index, indicating whether there is an ongoing inflammatory process, it was shown that there were no statistically significant differences between the analyzed parameters and the level of galectin-3. The Pearson correlation coefficient was used to assess the correlation between the analyzed variables. The only correlation that turned out to be statistically significant (*p* = 0.008) was the relationship between the concentration of creatinine kinase (CK) and the level of galectin-3 in people with SVT supraventricular arrhythmia. This correlation is very strong (R = 0.85), as shown in Figure 3.

## 4. Discussion

Tachyarrhythmia is a well-documented pediatric problem that can precipitate significant pathological processes, including death. The identification of biomarkers associated with paroxysmal or sustained tachycardia has implications for the early identification and effective treatment [15,16]. Galectin-3 has been identified as a potential biomarker that may be a predictor of prognosis and treatment in a variety of heart disease conditions [17,18,19,20]. Studies show that Gal-3 has a positive correlation with risk factors for the development of cardiovascular diseases, such as atherosclerosis and ischemic stroke [18,21,22].

In this study, the “zero” hypothesis assumed that there is a positive correlation between galectin concentration and cardiac arrhythmias in children. This is an extension of our research [2] with another molecular aspect. The study included 50 participants: 30 with a confirmed diagnosis of cardiac arrhythmia and 20 without arrhythmia as a control group. Statistical analysis performed on the entire group of patients, regardless of the type of cardiac arrhythmia, showed that there is no statistically significant difference in the value of Gal-3 concentration depending on gender. Therefore, based on our study, we cannot confirm a significant effect of gender on the value of Gal-3 concentration in the population of patients with cardiac arrhythmias.

However, some published studies showed a relationship between gender and Gal-3 concentration in the context of cardiac arrhythmias in adults. In a population study, Ho et al. [23] showed that the concentration of Gal-3 is significantly higher in women than in men among people not diagnosed with heart defects. Anand et al. [24] also showed that the concentration of Gal-3 is significantly higher in women than in men. In their study on a group of 59 patients with heart failure and healthy men and women (10 each), Schindler et al. [25] indicated no significant relationship between gender and the concentration of Gal-3, which is consistent with the results obtained in our study. Due to discrepancies in the results of various studies, further research is warranted to elucidate the relationship between gender and Gal-3 concentration in the context of cardiac arrhythmias.

In their study conducted on a group of 122 adult patients, Sanchis-Gomar et al. [26] showed a decrease in Gal-3 concentration with age. The discrepancy among these results may be attributed to variabilities in the size of the study group, as well as differences in the age of patients, given that our research concerns a group of children and adolescents.

Previous studies have highlighted the importance of galectin-3 as a biomarker in assessing the severity of cardiovascular diseases [5,7,8,10,13,16,17,20,25]. However, in the context of this study’s results, no significant relationship was observed between changes in galectin-3 concentration and the occurrence or worsening of specific clinical symptoms classified on the NYHA scale, regardless of the type of arrhythmia. This differs from the observations made by other research teams, e.g., Felker et al. [27], who observed that patients with elevated galectin-3 levels had features associated with an increased risk of heart failure, in which the concentration of galectin-3 was proportionally increasing together with the NYHA class [27].

Studies have shown that hypertension promotes left ventricular hypertrophy, which, in turn, increases the risk of ventricular arrhythmia [28]. Baron [28] also observed that QT prolongation was associated with a greater risk of developing unsustained ventricular tachycardia and complex ventricular arrhythmias in patients suffering from hypertension. This finding suggests that the analysis of the QRS parameter may have significant diagnostic and prognostic significance and be an indicator of the severity of cardiac arrhythmias [28]. However, our study did not demonstrate a relationship between total QRS and Gal-3 concentration. This confirms the need for further research on the use of the QRS parameter in identifying patients with arrhythmia and assessing the effectiveness of therapy and clinical prognosis.

The relationship between Gal-3 concentration and other echocardiographic parameters appears to be controversial. According to Chen et al. [29], a relationship does exist, e.g., between LVEDD (left ventricular end-diastolic diameter) and Gal-3, whereas Lisowska et al. [30] did not demonstrate such a correlation in adult patients, emphasizing that no relationship was observed between Gal-3 and the echocardiographic parameters used for analyzing the structure and functioning of the heart muscle (LVEDD, LVEF [Left ventricular ejection fraction], and LVMI [left ventricular mass index]). In our study, conducted on a group of pediatric patients, we also found no relationship between Gal-3 concentration and echocardiographic parameters. Furthermore, as previously indicated in the results section, no notable discrepancies were observed in the study group with regard to left ventricular systolic function, which was quantified as the left ventricular ejection fraction (EF).

Although many researchers emphasize the important role of Gal-3 in the prognosis, diagnosis, and assessment of the severity of ischemic heart disease (IHD) [10,31] and other cardiac diseases [18,20], the literature contains analyses presenting counter-arguments to the above considerations. For example, Jansen et al. [32] disputed that Gal-3 could be a predictor of cardiovascular events in patients with IHD, finding no relationships between the mentioned parameters. Moreover, the results presented in this study do not confirm the existence of significant relationships between the concentration of Gal-3 and heart diseases such as VT arrhythmia and SVT arrhythmia. As a result, the conducted research is not consistent with the views of other researchers who emphasize the significant role of Gal-3 in the prognosis and diagnosis of cardiovascular diseases 

For example, a study by Frogoudaki et al. [33] demonstrated that Gal-3 can be utilized as a marker of fibrosis in adult patients with congenital heart defects, exhibiting significantly elevated levels in the patient cohort. Furthermore, Gal-3 demonstrated a positive correlation with SVT and VT [33]. The difference between the results of this study and those obtained by Frogoudaki et al. [33] could result primarily from the age difference and idiopatic form of arrhythmia.

Some studies have investigated the correlation between galectin-3 concentration and the occurrence of atrial fibrillation (AF). It has been shown that the amount of the analyzed protein in plasma is greater in patients with persistent atrial fibrillation than in patients without the arrhythmia [11,19,34]. A simultaneous correlation was noted for metabolic syndrome and AF. Additionally, it has been reported that there is a relationship between the Gal-3 level and the duration of arrhythmia. The patients suffering from persistent arrhythmia exhibited lower Gal-3 concentrations compared to those with metabolic syndrome and permanent AF [35,36].

This study showed a strong relationship between the concentration of creatinine kinase and the level of galectin-3 in people with SVT supraventricular arrhythmia. Despite the lack of literature data on the relationship between these factors, it should be noted that both galectin-3 and creatinine kinase are mentioned by researchers as markers of heart failure and myocardial infarction [37,38]. High CK levels may also indicate myocardial damage [38,39], and Gal-3 is associated with cardiac inflammation and fibrosis [21,22].

The next issue is the relationship between the analyzed protein and blood thrombogenicity (tendency to thrombosis) in patients with AF. Patients with persistent arrhythmia have a positive correlation of Gal-3 with the presence of a thrombus in the left atrium during TEE (transesophageal echocardiography), as well as with the volume of LAA (left atrial appendage) [40]. Plasma Gal-3 concentrations exceeding 15 ng/mL translate into an increased risk of recurrence of persistent arrhythmia in patients after ablation [41,42]. However, the Gal-3 concentrations obtained in our study were lower than this value in the vast majority of patients. It is possible that the analyzed protein—in addition to the left atrial size—may become a marker of arrhythmia recurrence in the future, but this issue requires additional research [41,42].

Nonetheless, a positive relationship was observed between Gal-3 concentration and the development of ventricular arrhythmias, i.e., nsVT (non-sustained ventricular tachycardia) and sVT (sustained ventricular tachycardia), in people with ARVD (arrhythmogenic right ventricular cardiomyopathy). In a study by Oz et al. [43], it was demonstrated that serum Gal-3 levels, as determined via ELISA assay, exhibited a notable elevation in patients with ARVD. The findings suggested that serum Gal-3 levels may serve as a promising biomarker for the diagnosis of ARVD. However, they noted that the results of their research need to be verified in larger prospective studies. The results of our study were contradictory to those obtained by Oz et al. [43]; despite the similar size of the study group and the control group, our study did not demonstrate a relationship between Gal-3 concentration and the occurrence of SVT and VT arrhythmias.

When examining new markers of heart failure, it is also worth considering a comparison with existing markers already used for this purpose in clinical trials, such as NT-proBNP (N-terminal pro-B-type natriuretic peptide). The existing literature contains conflicting data regarding the diagnostic value of galectin-3 in comparison to NT-proBNP. Some studies have shown that Gal-3 may be a potential standalone marker of heart failure [23] and demonstrated an increase in the diagnostic value of Gal-3 as a marker when combined with the NT-proBNP concentration test [44]. As summarized by Schmitt et al. [45], a more detailed analysis and distinction in studies of different heart failure entities is required to identify a clinical application for galectin-3.

The recent study by Pietrzak et al. [46] found that the plasma concentration of galectin-3 is higher in teenagers with ventricular excess beats compared to healthy controls. This study shows a direct positive correlation between galectin-3 plasma level and left ventricular diastolic diameter and a reverse correlation between galectin-3 plasma level and left ventricular contractile function. In the multiple linear regression analysis, the CMR left ventricular ejection fraction and the number of arrhythmias were identified as two independent predictors of increased galectin-3 levels. Similarly to the other researchers, Pietrzak et al. acknowledged the potential for bias in their results, citing the possibility of confounding factors such as cardiac β-adrenergic receptor stimulation, which can influence the incidence and complexity of arrhythmias, as well as galectin-3 plasma concentration [46,47].

To sum up, despite existing reports suggesting a significant role of Gal-3 in the prognosis, diagnosis, and assessment of the severity of cardiac arrhythmias, this study did not demonstrate a significant correlation (relationship) of Gal-3 concentration with the occurrence and progression of VT or SVT arrhythmias. Notably, our study analyzed serum (unlike other studies, which focused on plasma). Additionally, our study group of patients included children suffering from the idiopathic form of arrhythmias—the most difficult to recognize in children. It must also be emphasized that to the best of our knowledge, only one study has been published in the available literature on the relationship between Gal-3 concentration and the occurrence of arrhythmia in children so far [46]. In this context, our study introduces new information regarding the usefulness of Gal-3 as a marker for arrhythmias in children. The limitation of this study is the small group of patients, considering the type of arrhythmia. All patients originated from one medical center. Further, all were children and adolescents, and as such, their galectin levels may also depend on other developmental factors. The study results are a valuable addition to the existing knowledge on the role of Gal-3 in cardiac arrhythmia.

## 5. Conclusions

The analyses conducted during this study made it possible to formulate the following conclusions: The level of galectin-3 in healthy children did not differ statistically from people diagnosed with cardiac arrhythmia. The level of galectin-3 is not dependent on the type of arrhythmia VT and SVT. The analysis did not show a significant relationship between gender, age, and galectin-3 concentration. The results of the study indicate a statistically significant relationship between the concentration of creatine kinase and the concentration of galectin-3 in patients with SVT-type arrhythmias, which suggests a potential influence of CK on the level of Gal-3. The results suggest that galectin-3 may not be a reliable diagnostic biomarker in the context of these specific types of arrhythmias, indicating a need for further research to identify other potential diagnostic markers for them. This is a prospective study. Furthermore, the discovery of additional innovative, non-invasive, sensitive, and reliable biomarkers is necessary to enhance the prognosis of idiopathic arrhythmias in children.

## Figures and Tables

**Table 1:** Basic characteristics of patients with arrhythmias and the control group. Holter ECG findings in the study group (the results are presented as the mean ± SD) [2,14].

Basic Characteristics	Study Group (*n* = 30)	Control Group (*n* = 20)	*p*-Value
Gender (F/M)	16/14	12/8	n/a
Mean age (years)	15.5 ± 1.9	15.8 ± 1.2	>0.05
BP syst (mmHg)	117.5 ± 10.4	120.5 ± 14.5	>0.05
BP diast (mmHg)	64.9 ± 8.0	62.6 ± 0.6	>0.05
Sat O_2_ (%)	98.3 ± 0.7	98.4 ± 1.1	>0.05
**Blood Laboratory tests**			
CK-MB (U/L)	14.1 ± 5.3	12.1 ± 5.8	>0.05
WBC (10^3^ u/L)	6.49 ± 1.83	6.88 ± 2.08	>0.05
RBC (10^6^ u/L)	4.88 ± 0.45	4.80 ± 0.47	>0.05
HGB (g/dL)	13.08 ± 1.31	14.0 ± 1.25	>0.05
HCT (%)	41.5 ± 2.7	41.1 ± 3.8	>0.05
TSH (mL U/L)	2.43 ± 1.05	2.36 ± 1.27	>0.05
FT4 (ng/dL)	1.3 ± 0.18	1.4 ± 0.39	>0.05
**Echocardiography parameters**			
LVIDD (mm)	48.7 ± 4.5	48.5 ± 3.1	>0.05
LVIDS (mm)	31.7 ± 13.7	28.8 ± 3.3	>0.05
LV-EF (%)	68.7 ± 7.9	69.9 ± 5.9	>0.05
LV-SF (%)	40.7 ± 6.1	39.9 ± 4.9	>0.05
**Exercise test**			
Stress test (gr)	3.7 ± 0.6	3.8 ± 0.5	>0.05
METS	11.2 ± 1.1	11.4 ± 0.73	>0.05
**24 h Holter ECG findings**	**Study group (*n* = 30)**	**Control group (*n* = 20)**	***p*-Value**
Number of QRS complexes/24 h	97.421 ± 1.534	98.089 ± 9.350	>0.05
Abnormal QRS mean (%); min-max	5.1 (1–30)	0	
HR mean	73.3 ± 0.9	72.7 ± 7.8	>0.05
HR max	123.3 ± 18.8	126.0 ± 13.6	>0.05
HR min	55.9 ± 1.3	52.7 ± 13.3	>0.05
**NYHA scale, *n***	**Study group (*n* = 30)**	**Control group (*n* = 20)**	***p* Value**
I	22	18	n/a
II	8	2	n/a
**Medications, *n*(%)**	**Study group (*n* = 30)**	**Control group (*n* = 20)**	***p* Value**
Class I antiarrhythmics	5 (16.7)	-	n/a
Class III antiarrhythmics	1 (3.3)	-	n/a
β-blockers	13 (43.4)	-	n/a
Calcium channel blockers	-	1 (5)	n/a
ACEi/ARB	1 (3.3)	1 (5)	n/a
Magnesium+B_6_	15 (50)	-	n/a
Antidepressant SSRI	1 (3.3)	-	n/a

Abbreviations: BP—systolic (syst) and diastolic (diast) blood pressure; CK-MB—creatine kinase-MB -myocardial band; ECG–electrocardiogram; FT4—free thyroxine; HCT—hematocrit/packed cell volume (PCV); HGB—hemoglobin; HR mean/min/max—heart rate mean, minimum, maximum; LV-EF—left ventricle ejection fraction; LVIDD and LVIDS—left ventricular internal diameter end diastole and end systole; LV-SF—left ventricular systolic function; METS—metabolic equivalents in exercise testing; n/a—not applicable, NYHA Classification—Stages of Heart Failure: Class I—no symptoms and no limitation in ordinary physical activity, e.g., shortness of breath when walking, climbing stairs, etc.; Class II—mild symptoms (mild shortness of breath and/or angina) and slight limitation during ordinary activity; QRS complex—QRS interval in ECG; RBC—red blood cells; Sat O_2_ (%)—oxygen saturation; TSH—thyroid stimulating hormone; WBC—white blood cells.

## Data Availability

The data are unavailable due to privacy and ethical restrictions. The data presented in this study are available upon request from the corresponding author.
